# Osteochondral allograft transplantation for articular humeral head defect from ballistic trauma

**DOI:** 10.1016/j.xrrt.2024.04.008

**Published:** 2024-05-06

**Authors:** Melissa Soderquist, Leslie Barnes

**Affiliations:** Temple University Hospital Department of Orthopaedic Surgery and Sports Medicine, Philadelphia, PA, USA

**Keywords:** Osteochondral allograft transplant, OCA, Humeral head, Full-thickness cartilage loss, Gunshot wound, Shoulder trauma

Extremity injuries from ballistic trauma are common^19^; however, most ballistic fractures are to the lower extremity and make up the primary focus of existing orthopedic literature.^12^^,^^17^ Orthopedic injuries as a result of ballistic trauma are heterogeneous in nature^16^ and outcomes are typically impacted by degree of bone loss, bone exposure, and the extent of soft tissue injury.^4^^,^^18^^,^^20^ There is a paucity of information regarding articular cartilage defects from penetrating trauma. Articular cartilage defects present a particular challenge in the glenohumeral joint for the orthopedic surgeon and patient.^7^ These defects are less frequently encountered in the glenohumeral joint and are typically the result of closed shoulder trauma, recurrent instability, or previous surgery.^25^ Healing capacity for these defects is limited.^26^ Treatment of glenohumeral articular cartilage defects is a challenge in part due to the anatomic and biomechanical nature of the glenohumeral joint itself, including the relatively thin articular cartilage present on the humeral head and the high shear stresses experienced during rotation.^26^

Multiple different procedures have been developed for these defects, focused on providing pain relief and restoring the articular surface. Osteochondral allograft transplantation (OCA), osteochondral autograft transplantation, débridement, microfracture, and autologous cartilage implantation (ACI)/matrix-associated ACI are viable options to treat patients with osteochondral defects; however, defect size and bone loss are among important considerations when selecting an appropriate treatment option. Articular cartilage defects have been extensively studied in the knee. Investigations have shown microfracture to be beneficial in lesions less than 2 cm^2^ without subchondral bone loss^26^; full-thickness cartilage lesions between 2 and 4 cm^2^ can be addressed using ACI or osteochondral autograft transfer. Lesions greater than 4 cm^2^ can be addressed using ACI or OCA.^14^

OCA was initially utilized by Erich Lexer in 1908 and has been regularly used in the United States for over 40 years.^5^ Advantages of OCA include its applicability to varying size defects with subchondral bone involvement, it does not require a border of healthy cartilage, it is not impacted by prior microfracture procedures, has no donor site morbidity, and it allows for faster rehabilitation. Disadvantages include its availability, cost, limited options in the instance of graft failure, and operative learning curve. OCA is indicated for young active patients with full-thickness focal lesions greater than 1 cm^2^ that cannot undergo other restorative procedures such as arthroplasty, ACI, or autograft transplantation procedures due to age, defect size, depth, and location. Contraindications include advanced osteoarthritis and chronic post-traumatic defects.^5^

Much of the current data for osteochondral allograft use in the shoulder are from patients with documented instability.^3^^,^^8^^,^^9^^,^^23^ This report presents the surgical technique and case of a patient with a full-thickness articular cartilage defect of the humeral head as the result of ballistic trauma with treatment using OCA.

## Materials and Methods

### History

**Figure 1:**
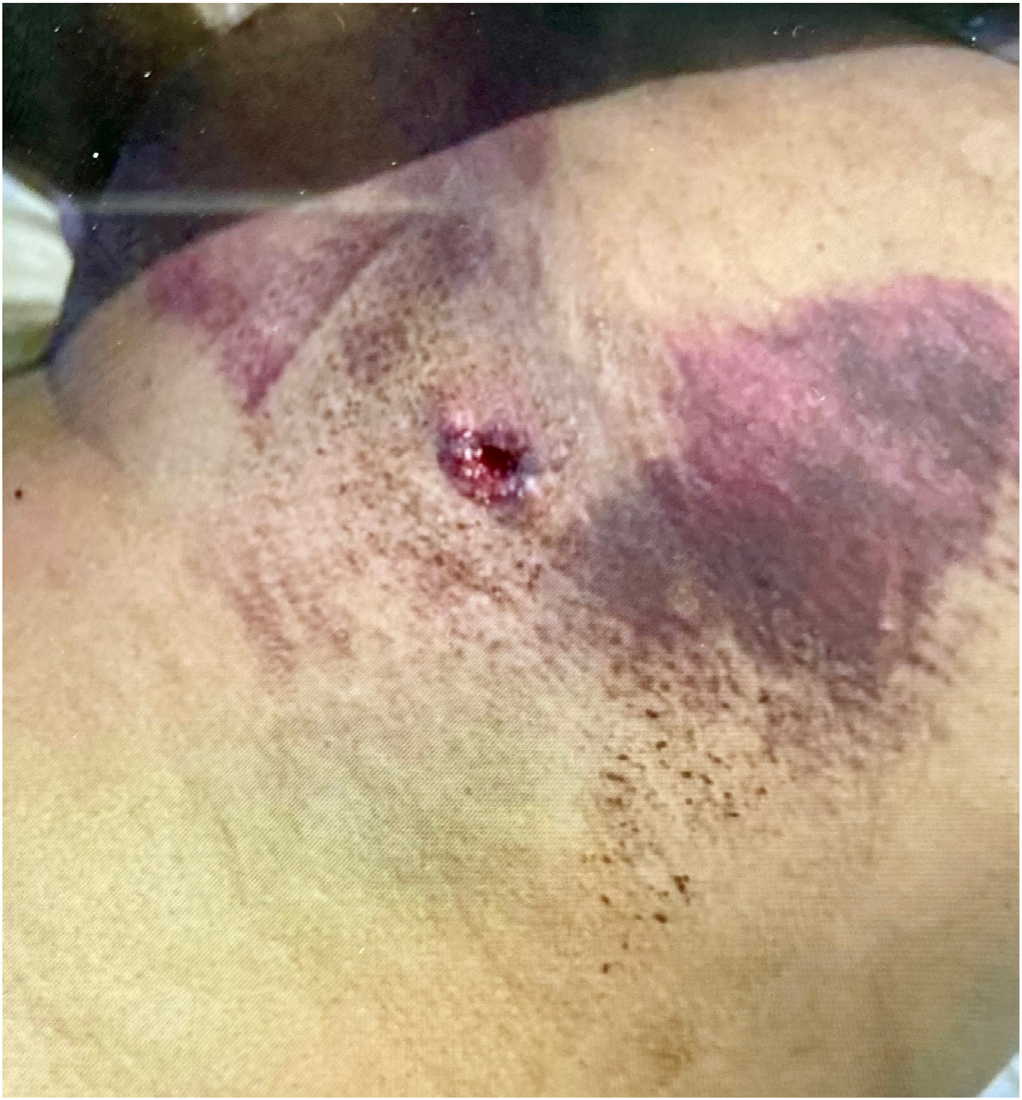
This image demonstrates the initial wound that the patient presented with to the emergency department, which is located at the *left* inferior aspect of the posterolateral axilla with the bullet trajectory directly into the axilla.

The patient in our case is a 41-year-old male who initially presented to the emergency department as a trauma evaluation with a gunshot wound to the left axilla (Fig. 1). He had a past medical history of cigarette smoking and opioid use. At the time of injury, he received 24 hours of intravenous antibiotics, tetanus prophylaxis and was placed in a sling. Computed tomography (CT) angiogram was negative for arterial injury. Imaging did confirm that he had sustained a traumatic arthrotomy to the glenohumeral joint with a retained 9-mm cylindrical missile in the humeral head located at the anterior humeral articular surface. He had no other injuries. He was hemodynamically stable and was discharged home, returning for his first evaluation as an outpatient in the shoulder and elbow clinic 6 weeks later. He reported significant left arm pain exacerbated by any movement and limited motion of the shoulder. He reported numbness and paresthesia throughout the left arm. He had tried Motrin and gabapentin for pain control without relief.

### Clinical examination

On clinical examination, there was a healed entry wound located at the left inferior aspect of the posterolateral axilla with a bullet trajectory directly into the axilla. There was no exit wound. The entry wound was located at the left inferior aspect of the posterolateral axilla with the bullet trajectory directly into the axilla. He was diffusely tender about the shoulder. Active and passive range of motion were both limited and equivalent in all planes with only 45° of forward flexion, 30° of external rotation and internal rotation to the sacrum. Shoulder strength was limited to 2 out of 5 on manual muscle testing, limited by pain. Active elbow, wrist, and hand range of motion was full. Sensation and motor were diminished in the axillary and musculocutaneous nerve distributions. His clinical examination was suggestive of mechanical symptoms from the retained foreign body and possible underlying brachial plexus pathology from the trauma.

### Imaging

**Figure 2:**
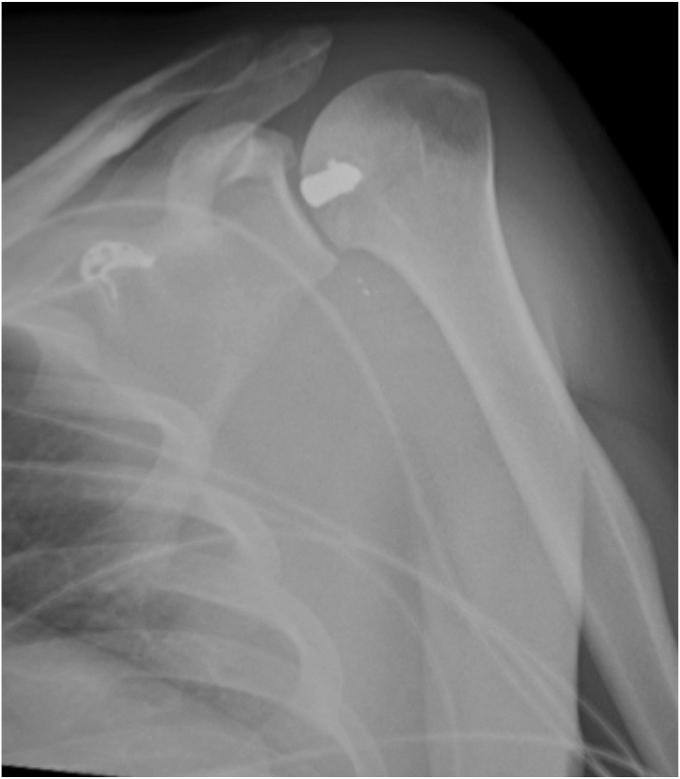
Initial injury radiograph, ballistic embedded in the humeral head.

**Figure 3:**
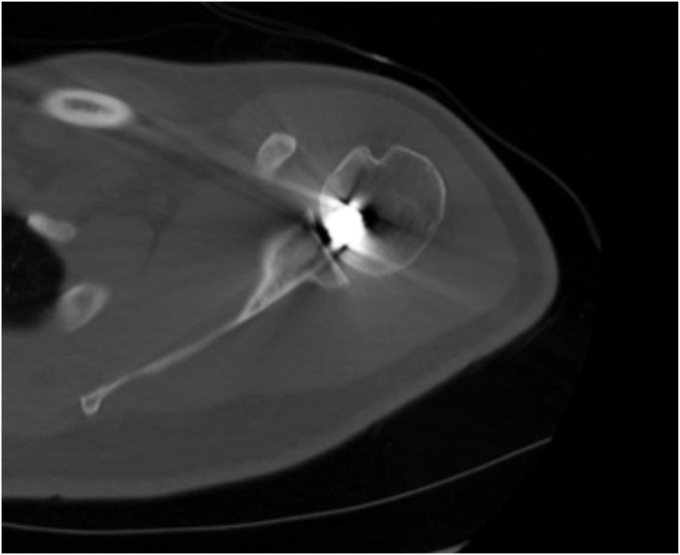
CT scan of retained missile in the humeral head. *CT*, computed tomography.

Radiographs were obtained and demonstrated a retained 9-mm cylindrical missile in the humeral head located at the anterior humeral articular surface with an associated nondisplaced humeral head fracture (Fig. 2). A dedicated shoulder CT scan (Fig. 3) was obtained which demonstrated the retained missile in the humeral head with protrusion into the glenohumeral joint. An electromyogram was initially planned as well, but the patient elected to proceed with surgical intervention without further delay due to his significant pain and limited motion. The procedure planned was a foreign body removal with OCA, with a preoperative diagnosis of a healed nondisplaced proximal humerus fracture with retained foreign body and osteochondral defect. Risks, benefits, and alternatives were discussed with the patient who elected to proceed. Written informed consent was obtained.

### Surgical technique

**Figure 4:**
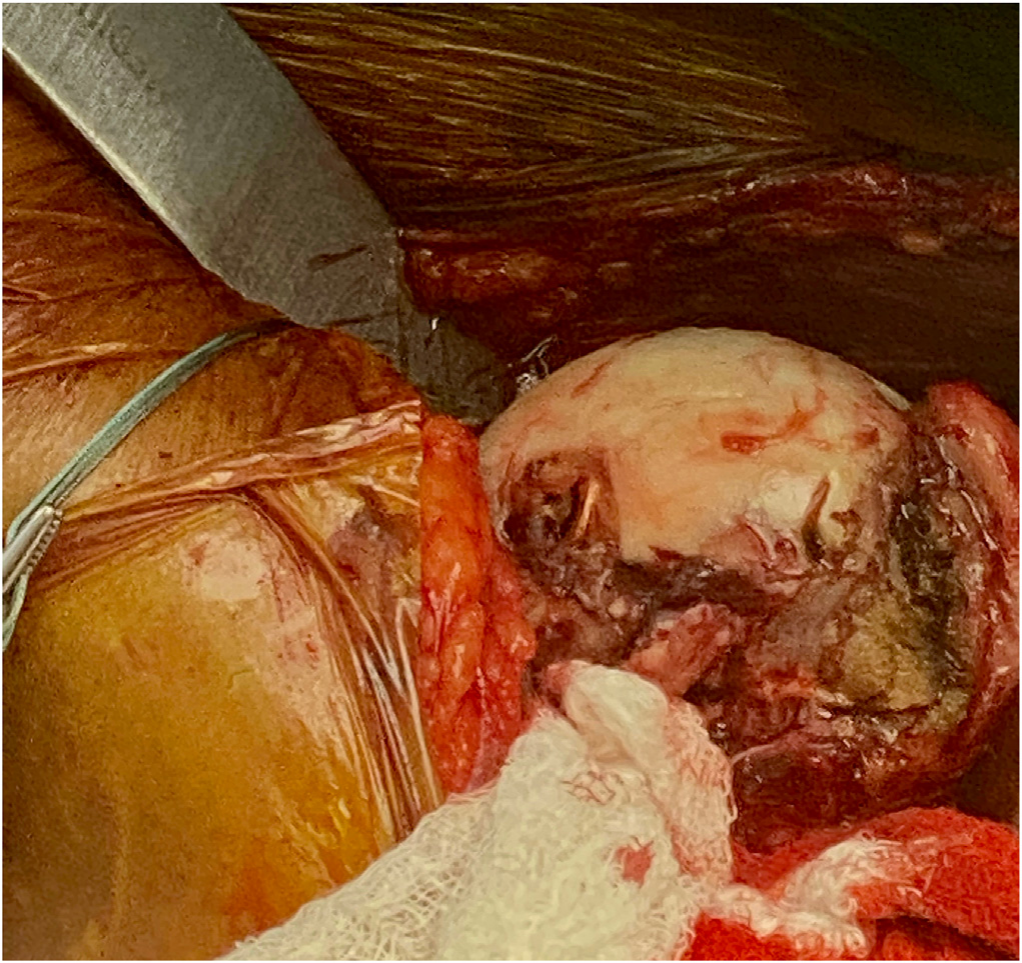
Foreign body protruding from the subchondral bone at time of surgery.

**Figure 5:**
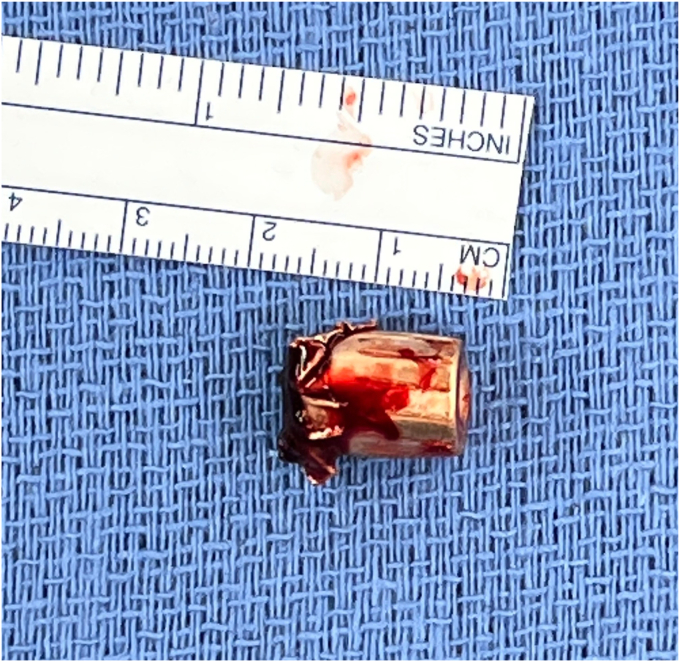
Extricated bullet in its entirety.

**Figure 6:**
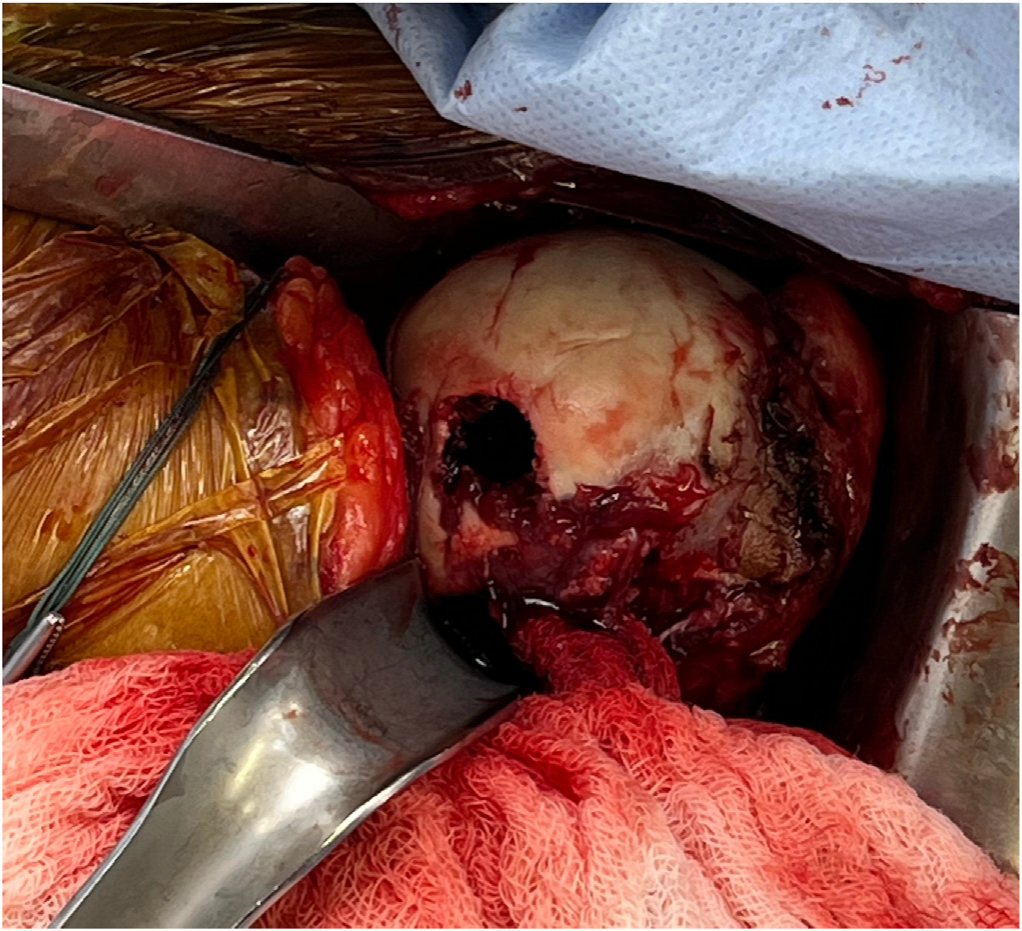
Humeral head osteochondral defect after bullet extrication.

**Figure 7:**
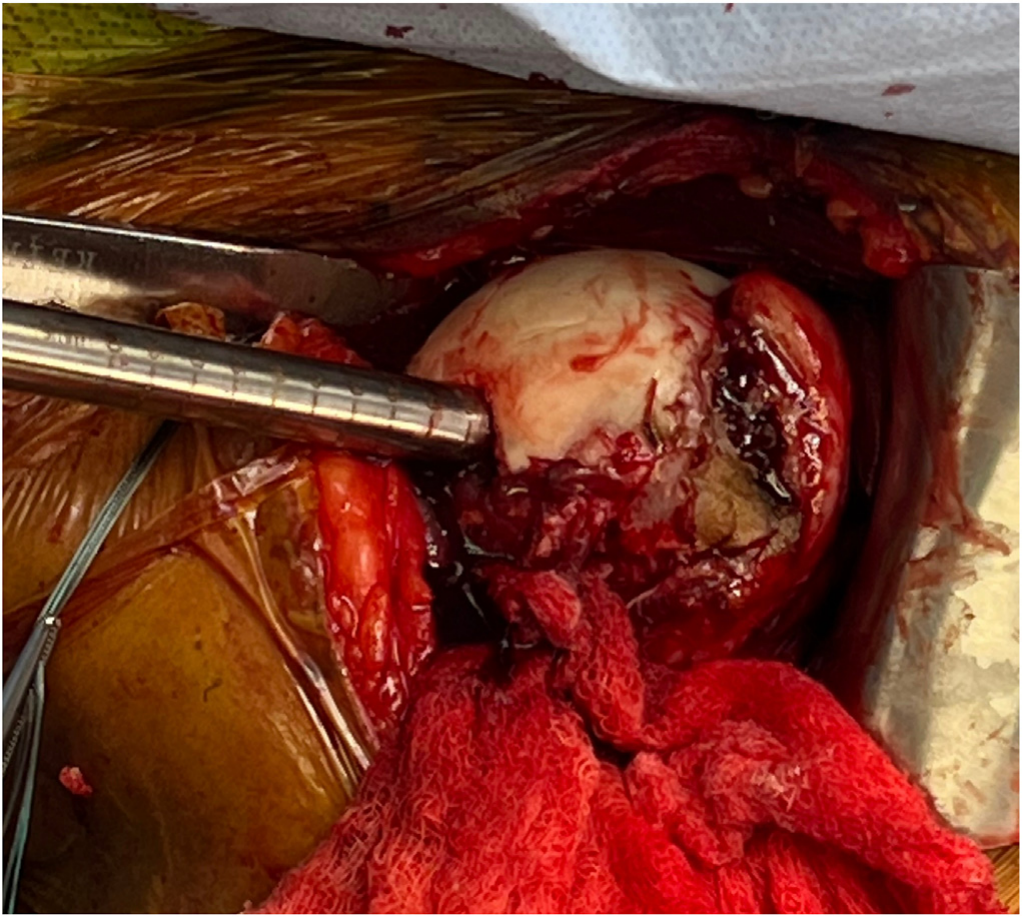
A 9-mm reamer used to clean debris from the osteochondral defect.

**Figure 8:**
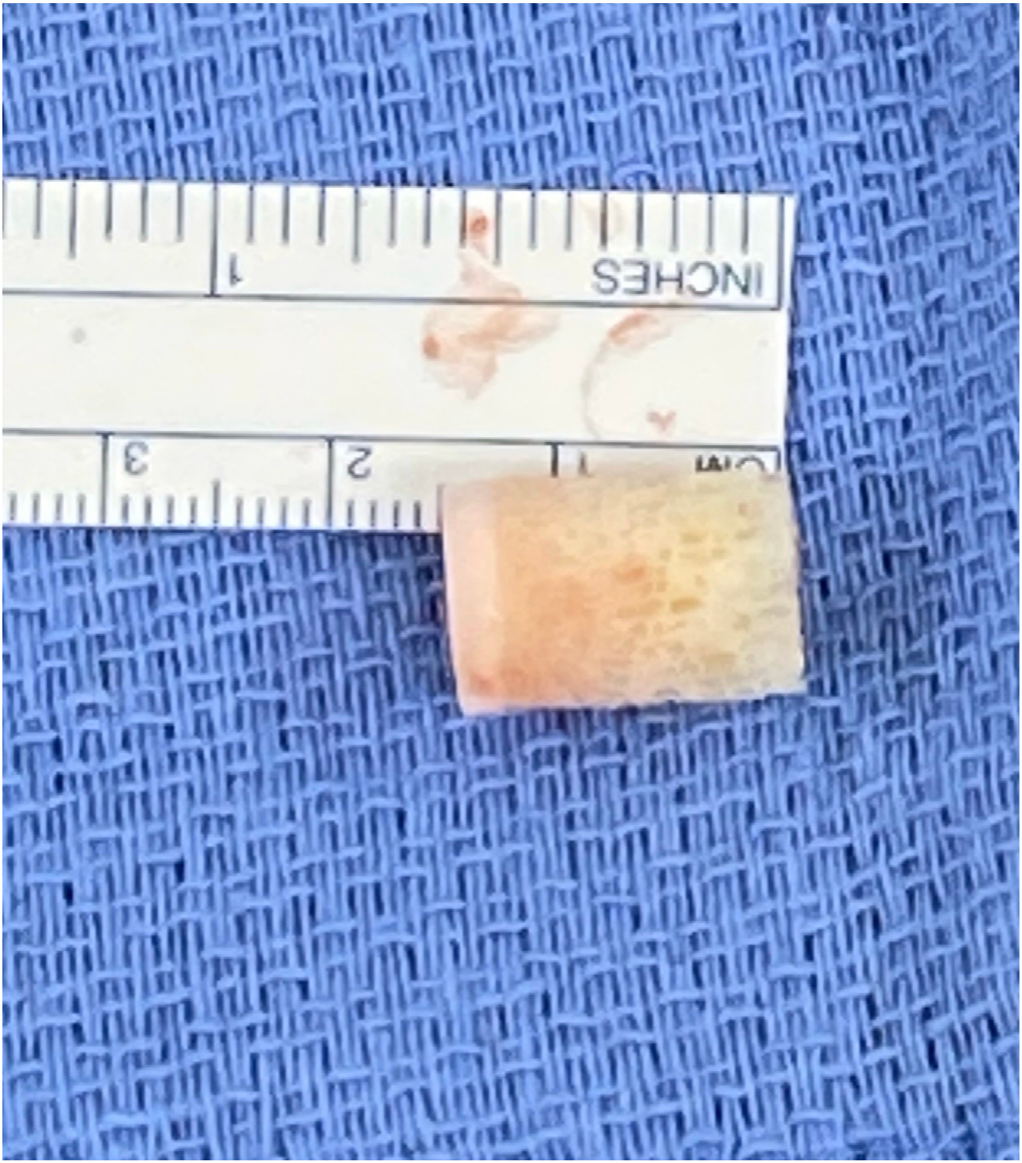
Fresh osteochondral allograft, 10 mm × 14 mm in size (Arthrex Inc., Naples, FL, USA).

**Figure 9:**
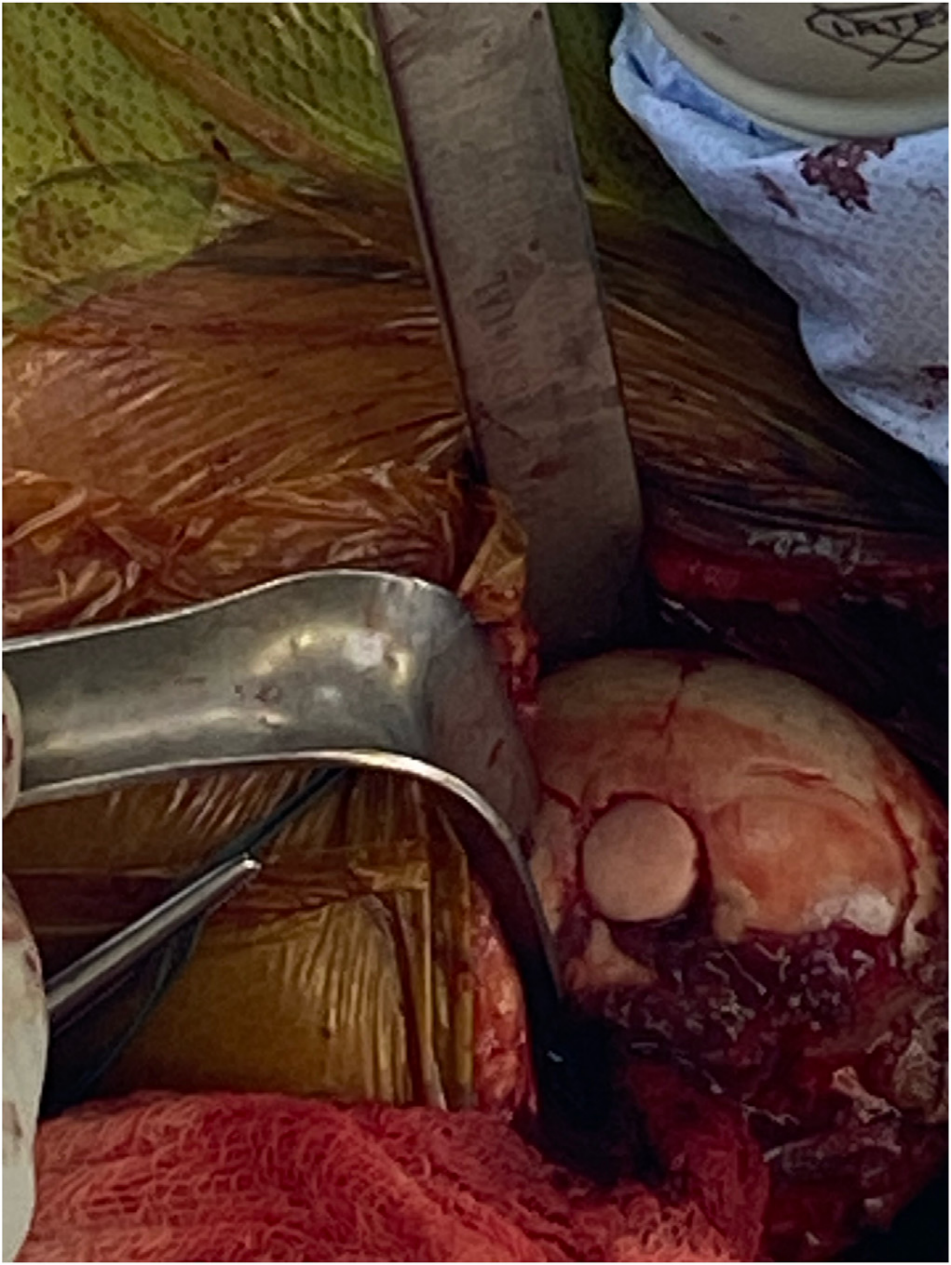
Osteochondral allograft press-fit into the osteochondral defect.

**Figure 10:**
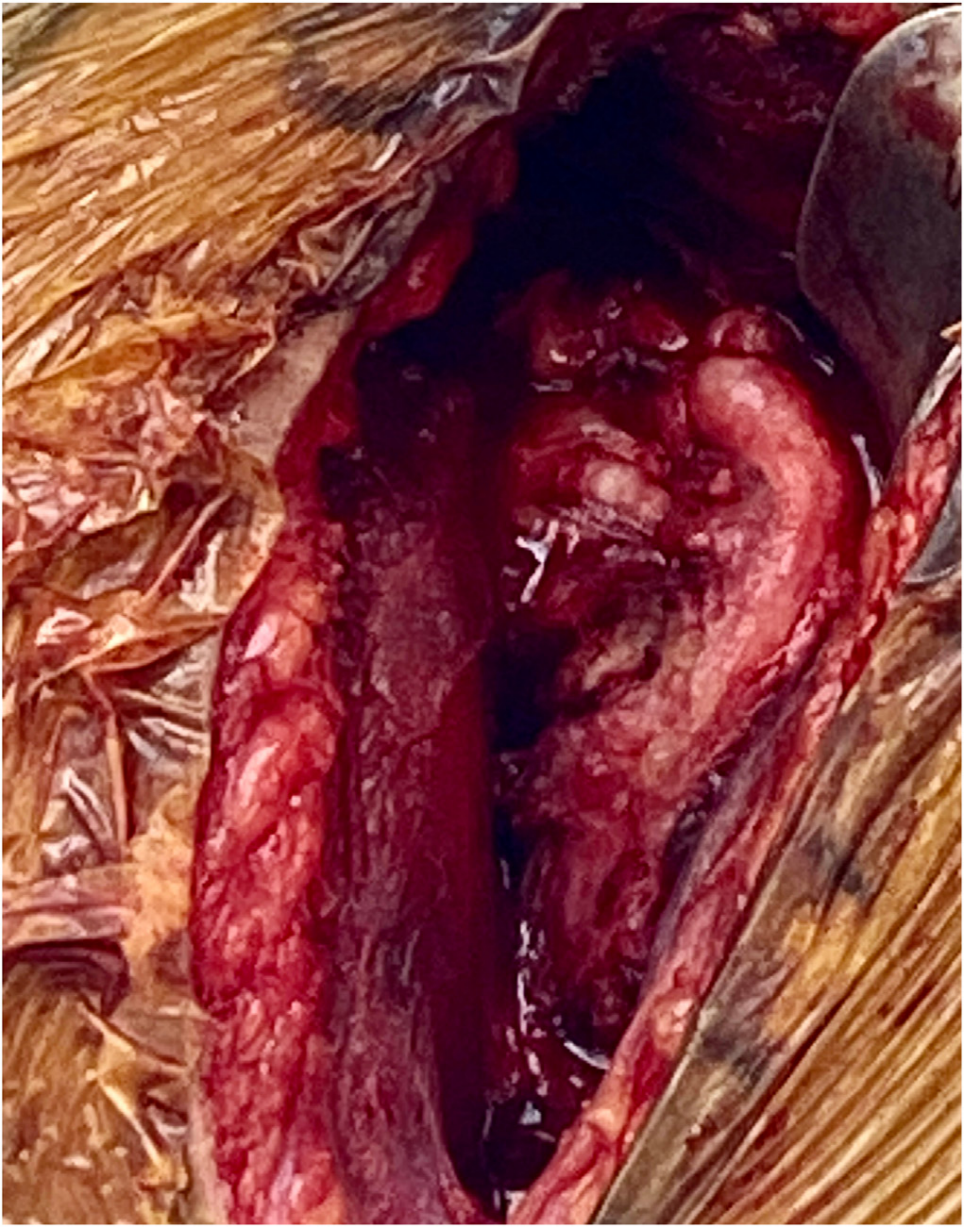
The subscapularis was repaired to bone using suture anchors and the upper border repair was reinforced with suture.

Before the procedure, he was offered a regional nerve block. However, he did decline this in light of his neurological symptoms and possible brachial plexus pathology. He was given general anesthesia and preoperative antibiotics per protocol. The patient was positioned in the semi-upright beach chair position and a pneumatic arm holder was used. The operative arm was prepped and draped in the usual sterile fashion. A limited deltopectoral approach was utilized. Subscapularis tenotomy was performed to gain full access to the humeral head and the tendon was tagged with Ethibond (J&J MedTech, New Brunswick, NJ, USA) suture for later repair. The shoulder was carefully dislocated and on inspection of the humeral head, the bullet was protruding from the subchondral bone (Fig. 4). There was no cartilage remaining in the area of the gunshot injury. The bullet was extricated intact using a curette and clamp. The extricated bullet and corresponding osteochondral defect were both measured to be 9 mm × 13 mm in size with a cylindrical shape (Figure 5, Figure 6, Figure 7). The defect was sized using the Arthrex OCA instrumentation. (Arthrex Inc., Naples, FL, USA) Gentle reaming was performed by hand at the defect with a 9-mm reamer to clear debris. The defect and glenohumeral joint were then irrigated with saline using pulse lavage. Precut, fresh osteochondral allograft cylindrical cores in sizes from 6 mm to 16 mm in diameter were available from Arthrex Inc. A precut, fresh 10-mm diameter osteochondral allograft cylinder was selected.^1^ The cartilage thickness on the osteochondral allograft plug measured approximately 1.5 mm. The allograft measured 14 mm in length (Fig. 8). The allograft plug may be cut shorter, but in this case that was not required since the depth of the defect was within 1 mm of the allograft length and some compression of the cancellous bone was expected with impaction of the graft. The osteochondral allograft plug was then thoroughly irrigated with pulse lavage. A tamp was then used to seat the bone plug into the defect. A sponge was used to protect the graft while it was manually tapped into place. The fit was secure, and the articular surface was as flush as possible with the remaining articular cartilage (Fig. 9). The shoulder was then reduced, and the subscapularis was repaired with 2 Fibertape (Arthrex, Naples, FL, USA) sutures in mattress fashion threaded through two Fibertape and two 4.75-mm SwiveLock anchors (Arthrex, Naples, FL, USA) anchors, which were placed into the lesser tuberosity. An additional Fiberwire (Arthrex, Naples, FL, USA) tendon-to-tendon suture was used to reinforce the upper border of the repair (Fig. 10). The shoulder was taken through a range of motion and was stable. The deltopectoral interval was then closed with absorbable 3-0 monocryl suture, followed by 3-0 monocryl sutures in the subcutaneous tissues and 4-0 monocryl running suture in the skin. The patient was placed in a sling with abduction pillow for the immediate postoperative period. Postoperative x-rays confirmed removal of the dominant bullet fragment, a filled defect with the osteochondral allograft and a concentric reduced glenohumeral joint.

## Results

**Figure 11:**
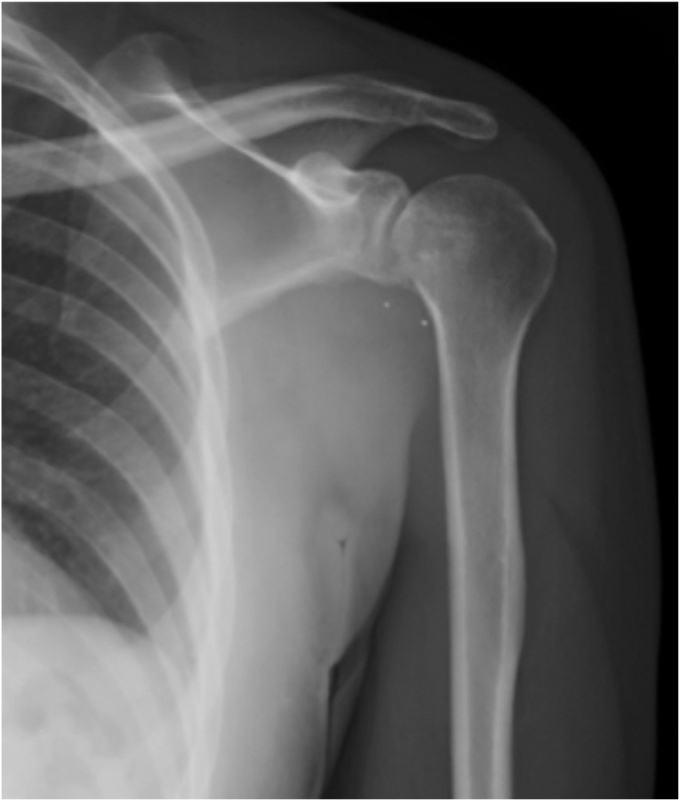
Stable appearing OCA on radiographic imaging at 2 weeks postoperative visit. *OCA*, osteochondral allograft transplantation.

**Figure 12:**
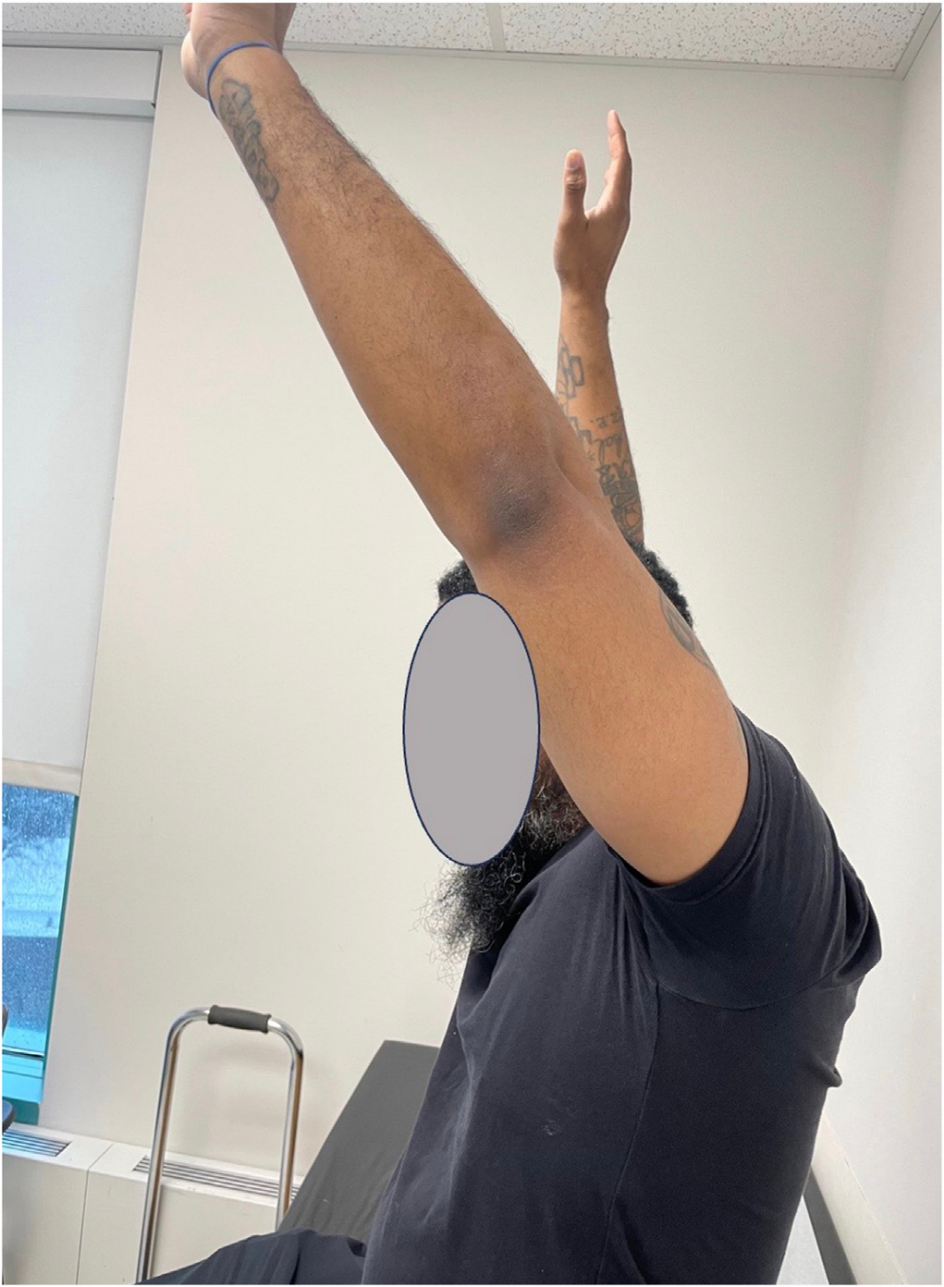
Active forward flexion at 20 months postoperative visit.

**Figure 13:**
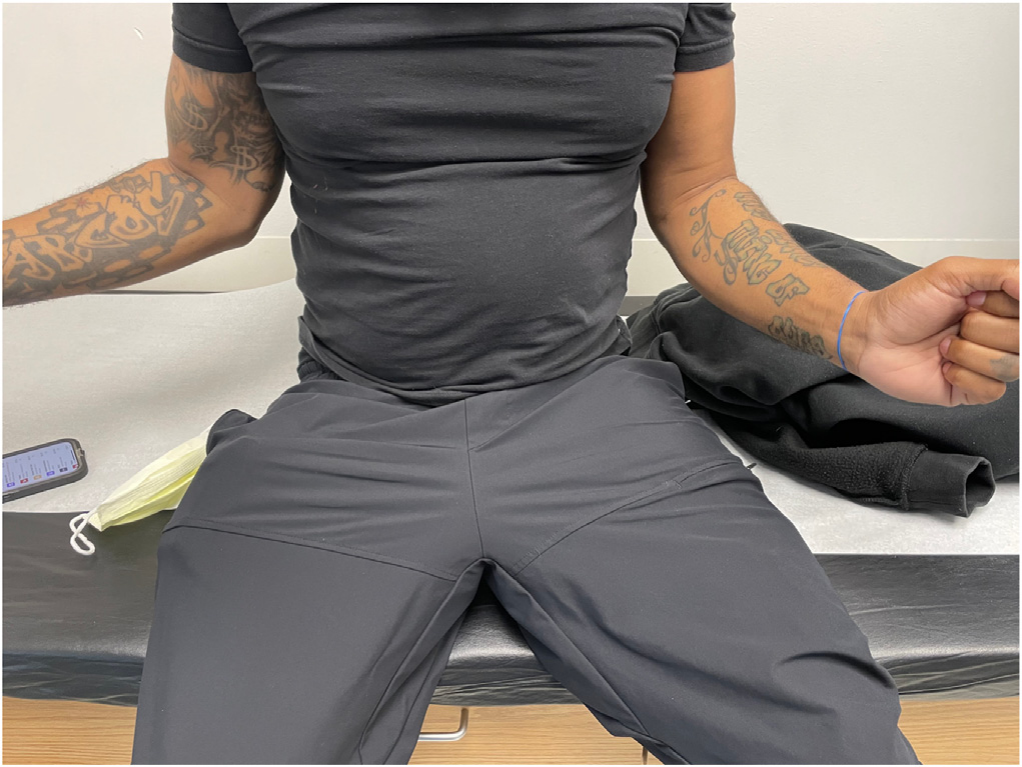
External rotation at 20 months postoperative visit.

**Figure 14:**
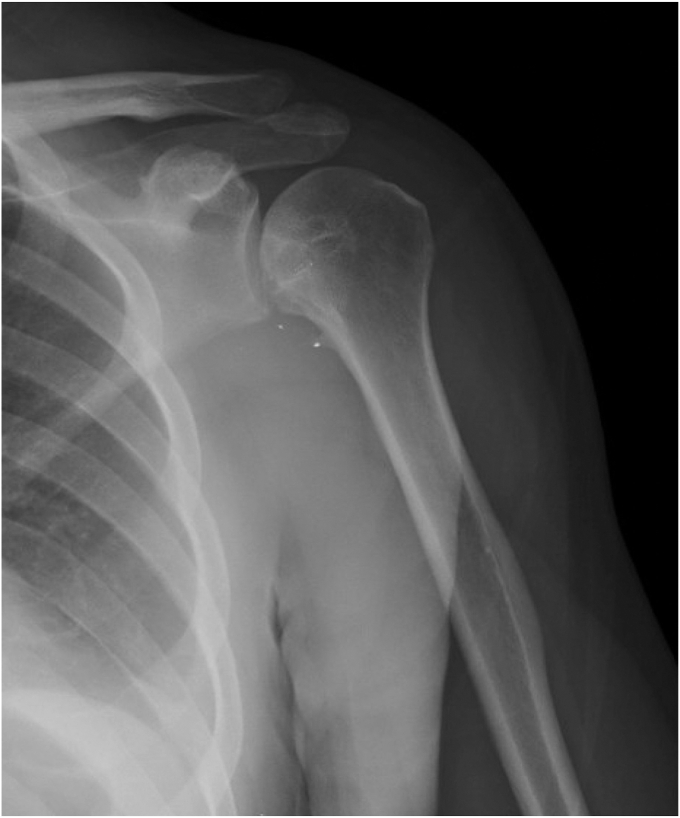
Stable, healed OCA on radiographic imaging at 20 months postoperative visit. *OCA*, osteochondral allograft transplantation.

At 2 weeks postoperatively, the patient had a stable appearing graft on radiographic imaging (Fig. 11) and was progressed to passive and active assist range of motion exercises with physical therapy. The prior fracture had fully healed. The sling was discontinued at 6 weeks postoperatively, and additional physical therapy was recommended. His neurologic symptoms also resolved. His graft continued to be stable and showed signs of incorporation. He was advanced to terminal stretches with physical therapy. He remained non–weight-bearing. At 3 and a half months postoperatively, his graft remained stable on radiographic imaging with good incorporation and he was progressed to weight-bearing as tolerated. At 6.5 months, his imaging remained stable and the glenohumeral joint was well preserved with no radiographic signs of arthrosis. His motion continued to improve although he had not been able to attend physical therapy. At 20 months postoperatively, he had returned to work as a laborer with 165° active forward flexion, abduction to 80°, external rotation to 70°, and internal rotation to T12 (Figs. 12 and 13). His strength was 5/5 and radiographs revealed stable, incorporated OCA (Fig. 14). The patient reported a Single Assessment Numeric Evaluation of 70.

## Discussion

Defect size and bone loss are 2 important factors in guiding treatment of these lesions. In focal lesions without bone loss; débridement, microfracture, OCA, and ACI/matrix-associated ACI are viable options. However, to adequately address patients with bone loss in addition to a cartilage defect, osteochondral allografts and joint resurfacing are the main options besides arthroplasty.^10^^,^^11^^,^^15^^,^^20^

OCA and ACI have been studied extensively in the knee and have been shown to be efficacious.^2^^,^^13^^,^^21^ These isolated defects are rare in the shoulder but there are currently data suggesting that OCA is as successful for glenohumeral articular cartilage defects as it is in the knee.^24^ There are currently limited data regarding osteochondral allograft use for penetrating trauma and ballistic injuries. Per the authors’ opinion, OCA is a valid option for patients with articular cartilage defects of the humeral head with associated bone loss from penetrating trauma.

Allograft use and specifically precut allograft, is advantageous in that it is a single-stage procedure, requires less operative time, is readily available, and has no donor site morbidity. Another potential benefit to osteochondral allograft is the possibility of implanting cartilage in better condition than that of the surrounding cartilage if from a younger donor. One of the downsides to fresh OCA is that it carries additional infection risk. The technique may be done open or arthroscopic assisted depending on the location and surgeon preference. In this case, the procedure was done open due to the location of the lesion. An alternative treatment option could include a bone dowel without overlying cartilage. This option is more sterilized as it is not a fresh graft, which is a benefit in reducing disease transmission; however, it lacks overlying cartilage. In our case, the size necessary was readily available and conveniently the same shape as the defect left from ballistic trauma. These osteochondral allograft cores are currently available in precut cylinders sized from 6 mm to 16 mm, which covers a wide range but still may not be the right shape or large enough for all defects. They may need to be combined to fill certain defects. Therefore, fresh humeral or femoral allograft would possibly be necessary for those defects. Previous investigation by Romeo et al has used osteochondral allograft cores for defects up to 30 mm in diameter with good results.^24^

Another potential downside is a cartilage thickness mismatch between the patient and the graft. The humeral head cartilage thickness at the center is estimated to be a mean of 1.2 mm, and near the periphery it is closer to 0.89 mm.^11^ The cartilage thickness on the allograft used was measured to be approximately 1.5 mm, which is slightly thicker than the local native anatomy.

Additionally, the shoulder experiences higher shear stress during rotation.^12^ Previous investigators have noted that chondral shear injuries and Hill-Sachs lesions among index anterior glenohumeral dislocations ranges from 47%-100% of cases.^23^ The long-term effects of these instability-related cartilage injuries are a precursor to glenohumeral osteoarthritis, especially when associated with continued instability.^23^ There are previous studies by Diklic et al and DiPaola et al that reported positive outcomes with regard to pain and activities of daily living for treatment of humeral head defects with OCA resulting from instability.^8^^,^^9^ Diklic et al managed 13 chronic posterior dislocations with defects in the humeral head involving 25%-50% of the articular surface with OCA. In that case series, the graft used was fresh-frozen femoral head osteochondral allograft and in contrast to our case, the graft required fixation with several screws due to the defect size as opposed to our press-fit. Constant scores, pain, strength, and activities of daily living were improved in these patients.^8^ DiPaola treated 4 patients with Hill-Sachs lesions with OCA and had no episodes of recurrent instability and good functional results.^9^ In this case, the humeral head defects were between 33% and 40% of the articular surface and were either treated using an open procedure with femoral head allograft or arthroscopically using osteochondral allograft plugs which had to be cut to size intraoperatively. The femoral head allograft required fixation with screws, similar to the study by Diklic.

Follow-up duration in this study exceeds published timelines of expected graft incorporation. Cook et al obtained a CT arthrogram at an average of 5 months postoperatively for 18 military patients who underwent OCA, and they created a grading system with which to evaluate both bone integration and cartilage congruency based on contrast distribution. The majority of patients had grade 1 (complete bone integration and flush articular surfaces) or 2 (bone cyst or defect <2 mm and single articular cartilage fissure without obvious defect) bone integration and cartilage at an average of 5 months postoperatively.^6^ With regard to our patient, his OCA did appear completely incorporated by 6 months and has been stable up until the study duration of 20 months follow-up.

In our case in particular, the missile was in a specific location due to the penetrating trauma sustained at the time of injury. The resulting defect was the size of the retained missile. The most common type of ammunition used in civilian ballistic injuries is 9 mm, which is what we found in our patient.^22^ In other cases of cartilage defect due to closed trauma, the cartilage lesion is typically shallower and the operative surgeon can choose the axis in which to core out the subchondral bone with respect to the arc of curvature of the surrounding articulation. The defect in our patient was a nearly perfect match for the osteochondral allograft plug selected to fill the defect. The benefits of this technique include the applicability to varying defect sizes, no donor site morbidity, and faster rehabilitation. This type of OCA is also advantageous in that there is no delay in obtaining a size matched articular allograft donor in certain situations where a cylindrical allograft would be suitable, such as in our case with ballistic trauma.

## Conclusion

These cartilage defects can occur in the shoulder or any articulation. There are limited data regarding OCA for ballistic injury. In our patient, the OCA procedure restored the humeral articulation with great improvement in motion, no mid-term complication, and solid graft incorporation at 6 months. With the rising incidence of firearm injuries and advances in cartilage restoration, further consideration should be given to this technique for similar injuries.

## Disclaimers:

Funding: No funding was disclosed by the authors.

Conflicts of interest: The authors, their immediate families, and any research foundations with which they are affiliated have not received any financial payments or other benefits from any commercial entity related to the subject of this article.

Patient consent: Written informed consent was obtained to take and publish clinical photographs and data.

## References

[1] (2018). Arthrex Inc. precut fresh osteochondral allograft cores. Product Manual.

[2] Bentley G., Biant L.C., Carrington R.W., Akmal M., Goldberg A., Williams A. (2003). A prospective, randomised comparison of autologous chondrocyte implantation versus mosaicplasty for osteochondral defects in the knee. J Bone Joint Surg Br.

[3] Boehm E., Minkus M., Scheibel M. (2020). Autologous chondrocyte implantation for treatment of focal articular cartilage defects of the humeral head. J Shoulder Elbow Surg.

[4] Bowyer G.W., Rossiter N.D. (1997). Management of gunshot wounds of the limbs. J Bone Joint Surg Br.

[5] Cavendish P.A., Everhart J.S., Peters N.J., Sommerfeldt M.F., Flanigan D.C. (2019). Osteochondral allograft transplantation for knee cartilage and osteochondral defects: a review of indications, technique, rehabilitation, and outcomes. JBJS Rev.

[6] Cook C.J., Shaha C.J., Rowles C.D., Tokish C.J., Shaha S.H., Bottoni C.R. (2015). Utility of computed tomography arthrograms in evaluating osteochondral allograft transplants of the distal femur. J Surg Orthop Adv.

[7] Depalma A.A., Gruson K.I. (2012). Management of cartilage defects in the shoulder. Curr Rev Musculoskelet Med.

[8] Diklic I.D., Ganic Z.D., Blagojevic Z.D., Nho S.J., Romeo A.A. (2010). Treatment of locked chronic posterior dislocation of the shoulder by reconstruction of the defect in the humeral head with an allograft. J Bone Joint Surg Br.

[9] DiPaola M.J., Jazrawi L.M., Rokito A.S., Kwon Y., Patel L., Pahk B. (2010). Management of humeral and glenoid bone loss--associated with glenohumeral instability. Bull NYU Hosp Jt Dis.

[10] Elser F., Braun S., Dewing C.B., Millett P.J. (2010). Glenohumeral joint preservation: current options for managing articular cartilage lesions in young, active patients. Arthroscopy.

[11] Fox J.A., Cole B.J., Romeo A.A., Meininger A., Williams J., Glenn Jr R.E. (2008). Articular cartilage thickness of the humeral head: an anatomic study. Orthopedics.

[12] Hahn M., Strauss E., Yang E.C. (1995). Gunshot wounds to the forearm. Orthop Clin North Am.

[13] Hangody L., Füles P. (2003). Autologous osteochondral mosaicplasty for the treatment of full-thickness defects of weight-bearing joints: ten years of experimental and clinical experience. J Bone Joint Surg Am.

[14] Matthews J.R., Brutico J.M., Abraham D.T., Heard J., Tucker B., Tjoumakaris (2022). Differences in clinical and functional outcomes between osteochondral allograft transplantation and autologous chondrocyte implantation for the treatment of focal articular cartilage defects. Orthop J Sports Med.

[15] McCarty L.P., Cole B.J. (2005). Nonarthroplasty treatment of glenohumeral cartilage lesions. Arthroscopy.

[16] Meade A., Hembd A., Cho M.J., Zhang A.Y. (2021). Surgical treatment of upper extremity gunshot injures: an updated review. Ann Plast Surg.

[17] Mehta S.K., Dale W.W., Dedwylder M.D., Bergin P.F., Spitler C.A. (2018). Rates of neurovascular injury, compartment syndrome, and early infection in operatively treated civilian ballistic forearm fractures. Injury.

[18] Metcalf K.B., Smith E.J., Wetzel R.J., Sontich J.K., Ochenjele G. (2020). Comparison of clinical outcomes after intramedullary fixation of tibia fractures caused by blunt trauma and civilian gunshot wounds: a retrospective review. J Orthop Trauma.

[19] Naidoo S. (2005).

[20] Omid R., Stone M.A., Zalavras C.G., Marecek G.S. (2019). Gunshot wounds to the upper extremity. J Am Acad Orthop Surg.

[21] Page R.S. (2008). Managing chondral lesions of the glenohumeral joint. Int J Shoulder Surg.

[22] Rhee P.M., Moore E.E., Joseph B., Tang A., Pandit V., Vercruysse G. (2016). Gunshot wounds: a review of ballistics, bullets, weapons, and myths. J Trauma Acute Care Surg.

[23] Riff A.J., Yanke A.B., Shin J.J., Romeo A.A., Cole B.J. (2017). Midterm results of osteochondral allograft transplantation to the humeral head. J Shoulder Elbow Surg.

[24] Romeo A.A., Cole B.J., Mazzocca A.D., Fox J.A., Freeman K.B., Joy E. (2002). Autologous chondrocyte repair of an articular defect in the humeral head. Arthroscopy.

[25] Seidl A.J., Kraeutler M.J. (2018). Management of articular cartilage defects in the glenohumeral joint. J Am Acad Orthop Surg.

[26] Weber A.E., Locker P.H., Mayer E.N., Cvetanovich G., Tilton A., Erickson B. (2018). Clinical outcomes after microfracture of the knee: midterm follow-up. Orthop J Sports Med.

